# Cases Extracted, by Permission, from the Note-Books of Henry Davies, M.D.

**Published:** 1834-01-01

**Authors:** 


### ORIGINAL COMMUNICATIONS.

*Cases extracted, by permission, from the Note-books of* HENRY DAVIES, M.D., Physician-Accoucheur to the Brownlow-street Lying-in Hospital, &c.

NO. II.

CASE I. Sept. 24th, 1832. Caroline Tiller, æt. twenty-four, reports that, two years ago, she had a slow recovery after a tedious labour, in which the child was still-born. Since this period she has had violent pain in the pubic region extending to the loins. She also complains of nervous head-ach, relaxed bowels, and a frequent desire of making water. The catamenia appear at irregular intervals, but oftener and in greater quantity than when she is in health: at these periods she suffers considerable pain. The catheter was passed, and the following medicines prescribed: ℞. Mist. Camph. lb ss.; Tr. Hyoscyami, Sodæ Carb. *aa* ʒj. M. sumat coch. iij. majora t. d. ℞. Pil. Saponis c̄ Opio, gr. ijss.; Hydr. Subm. gr. i. M. ut fiat pil. o. n. sum.

Sept. 28th. The diarrhœa is unabated, but she makes water less frequently, and the pain is less violent.—℞. Opii, gr. ss.; Hydr. Subm. gr. i. M. ut fiat pil. o. n. sum. Rept. mistura. The catheter was again passed.

Oct. 1. Is improving.

Oct. 5. Improvement continues. The catheter was again passed. She expects the catamenia to appear on the 15th.

Oct. 12th. ℞. Extr. Stramonii, Hydr. Subm. *aa* gr. i. M. ut fiat pil. o. n. per tres vices sumenda. Applicentur hirud. vj. regioni inguin.

℞. Pulv. Ipecac. c., gr. xx.; Extr. Stram. gr. i.; Extr. Hyoscyami, gr. xx. fiat pil. xij. sumat ij. quartis horis, si dolor persstiterit fluentibus catameniis. Semicupium.

Oct. 21st. The catamenia have appeared, and she has suffered less pain; she makes water seldomer, and is better altogether.

Oct. 26th. Is still improving.—℞. Inf. Cascar. ℥viij.; Sodæ Carb. ℈i.; Tr. Gran. Parad.* ʒss. M. sumat quartam partem bis in die. ℞. Pil. Rhei c.,† Extr. Hyoscyami *aa* ℈ss. M. fiat pil. vj. sumat i. o. n.

* The Tr. Granorum Paradisi, used at the Welbeck-street Dispensary as a cheap and pungent substitute for the tincture of cardamoms.

† A formula of the Edinburgh Pharmacopœia.—ED. *Med. Quart. Review.*

She continued to get better, and was discharged cured on the 12th of November.

Case ii. July 24th, 1820. Margaret Richardson, æt. forty-five, and the mother of two children, gives the following account of her complaints. Three years ago she had an attack of erysipelas, affecting her head and throat; it was attended with delirium, and followed by violent menorrhagia, which lasted several weeks. Since this time she has been subject to a vaginal discharge. The catamenia appear every second or third week, and continue from seven to ten days. The abdomen is enlarged as from ascites, the legs anasarcous, the countenance cadaverous. Her bowels are costive, her tongue and lips pale, and the eyes of a pearly white colour.—℞. Pil. Galbani c., Pil. Aloes c̄ Myrrhâ, *aa* gr. v. M. ut fiant pil. ij. o. n. sumendæ. ℞. Tr. Ferri Muriatis, ʒiss.; Acid. Nitr. dil., ʒviss. M. sumat coch. i. min. ex Inf. Zingib. bis in die.

She was ordered to use nutritious diet, and moderate exercise; to employ friction of the legs, and to bathe them in salt and water.

August 15th. Is improving.—℞. Hydr. Subm. gr. i.; Elaterii, gr. ¼; Aloes pulv., Cambog. pulv. *aa* gr. i.; Syrupi q. s. M. ut fiat pil. bis in septimanâ sumenda.

September 4th. Is much better, the abdomen being nearly of the natural size. The catamenial flow lasted only five days, and was not so violent as usual.

September 11th. The patient complains of violent pain in her right hip, but is better in other respects: her legs are of their natural size.

September 20th. Still improving.—Sumat Cinch. pulv. ʒss. bis in die, et Tr. Lyttæ, gtt. xxx. bis etiam in die. Pil. purg. p. r. n.

October 18th. Has been gradually mending till yesterday, when she had an attack of diarrhœa and menorrhagia.—Sumat statim Haust. Rhei cum Tr. Opii. ℞. P. Ipecac. c., Rhei pulv. *aa* gr. v. M. ut fiat pulv. h. s.s.

October 30th. The diarrhœa has been relieved, but she is much debilitated. She was ordered to take Infusion of Calumba, and the powder last prescribed was repeated.

November 20th. Discharged relieved.

This woman enjoyed tolerable health for a short time; but the cold of winter, and indifferent diet, overwhelmed her shattered constitution, and compelled her to apply again for relief in February, 1821. She was then suffering from anasarca, and, as her health required a nurse, which her circumstances could not afford, she was presented with a ticket for the Middlesex Hospital.

*Two Cases of Vascular Thickening of the Orifice of the Urethra.*

Case i. August 2d, 1820. Sarah Wise, aged thirty-seven, the mother of three children, who menstruates regularly, but suffers from leucorrhœa, complains of something which impedes the flow of urine. This obstruction is attended with occasional rigors, throbbing, bearing down, and a sensation as if a bladder of water were in the passage. She says that she has been treated for stone at one hospital, and for cancer at another. The action of the bowels is irregular, and the urine turbid; she sleeps ill, and suffers from sympathetic affection of the stomach. On examination, the vagina was found to be healthy, and there was no procidentia uteri; and, though the uterus was somewhat enlarged, it was not sufficiently so to constitute disease. There was a vascular thickening and enlargement of the orifice of the urethra, which was exquisitely sensible to the touch.

The diseased point was touched with the sulphate of copper, and a large bougie was passed; the patient was likewise ordered to touch the part, before she made water, with the Ung. Opii, and the following medicines were prescribed: Sumat Haust. Sennæ statim. ℞. Sodæ Carb. ℈i.; Glycyrrhizæ pulv. ℈ss. M. ut fiat pulvis bis in die sum. ℞. Hydr. Subm. gr. i.: Extr. Hyoscyami, gr. iij. M. ut fiat pil. o. n. sum.

September. Her general health is considerably improved, and she makes water much less frequently. She suffers a good deal from the application of the caustic, but is always better for a day or two afterwards.

October 12th. The patient is extremely grateful for the amendment of her health, and observes, that she is not so well if the bougie is not passed. She was therefore ordered to continue its use, as well as that of the Cupri Sulph., the Ung. Opii, and an occasional aperient.

November 6th. She feels much better, and the vascularity has almost disappeared.

November 20th. Nearly well.

December 4th. The bougie and the sulphate of copper are now used once a week only. The vascularity has entirely disappeared.

January 4th, 1821. The patient is scarcely sensible of any ailment, but continues the occasional use of the bougie.

February 25th. She continues free from complaint.

Case ii. May 4th, 1821. Elizabeth Leaver, æt. forty-three, has generally had good health, but has never borne children. She complains of a vaginal discharge, for which she has been under treatment by two surgeons for several months: since its appearance she has not cohabited with her husband. On examination, the os externum was found to be much contracted, so that the insertion of the finger caused great pain; the uterus and vagina were healthy; but there was an enlargement at the commencement of the urethra, the orifices of the mucous ducts being ulcerated, and the contiguous parts inflamed.—Applicetur Cupri Sulph. orificio urethræ ter in septimanâ. Laventur partes dolentes aquâ frigidâ bis in die. Sumat Pil. aperientes, iij. p. r. n.

May 30th. The ulcers are healing, and the vascularity of the orifice of the urethra is diminishing. A large bougie was passed, with the intention of diminishing the extreme sensibility of the part, and allowed to remain ten minutes.—Continueter usus Cupri Sulph.

June 30th. The patient says that she can make water with ease, and that the irritability of the parts has ceased; but the os externum is still too much contracted to permit cohabition. She was ordered to inject tepid water into the vagina from a large syringe three times a day.

August. The patient (who had returned to her husband's bed,) was discharged cured.

This woman was seen some months afterwards (November 10th,) in the enjoyment of perfect health.

